# Perceived reward attainability may underlie dogs’ responses in inequity paradigms

**DOI:** 10.1038/s41598-023-38836-w

**Published:** 2023-07-26

**Authors:** Jim McGetrick, Hugo Peters, Anna D. J. Korath, Romana Feitsch, Susanne Siegmann, Friederike Range

**Affiliations:** 1 Domestication Lab, Konrad Lorenz Institute of Ethology, Department of Interdisciplinary Life Sciences, University of Veterinary Medicine, Vienna, Dörfles 48, 2115 Ernstbrunn, Austria; 2 Institute of Animal Welfare Science, University of Veterinary Medicine, Veterinärplatz 1, 1210 Vienna, Austria

**Keywords:** Psychology, Social evolution, Animal behaviour, Social behaviour

## Abstract

Dogs have repeatedly been shown to give their paw to an experimenter more times for no reward when a rewarded conspecific partner is absent than when a rewarded conspecific is present, thereby showing inequity aversion. However, rather than being inequity averse, dogs might give their paw more when a partner is absent due to the experimenter’s procedure in which they move food in front of the subject to mimic feeding a partner. This action could increase subjects’ perception of reward attainability. We tested this hypothesis by introducing an improved type of control condition in which subjects were unrewarded for giving the paw in the presence of a rewarded box, a condition that more closely resembles the inequity condition. Inequity averse subjects’ performance did not differ based on whether the partner was another dog or a box. Moreover, these subjects gave the paw more times when no partner was present and the experimenter mimicked the feeding of a partner than when rewards were placed in the box. These results suggest that responses in the previous studies were inflated by subjects’ increased perception of reward attainability when no partner was present and, therefore, over-exaggerated dogs’ propensity to give up due to inequity aversion.

## Introduction

Many non-human animal species have been shown to display a negative behavioural response to (disadvantageous) inequity in experimental contexts^1,2^. This response, commonly referred to as inequity aversion and considered to reflect social comparison of payoffs, has attracted considerable attention due to its putative involvement in the evolution of a sense of fairness and the stabilization of cooperation^1,3–6^. However, skepticism and controversy persist over reports of inequity aversion in non-human animals, particularly primates, due to a variety of proposed alternative explanations for observed responses^7–13^ (but see also Brosnan^14^ and van Wolkenten et al.^15^), as well as numerous failed replications across research groups^16–21^ (but see also Brosnan et al.^22^, Hopper et al.^23^, and Fletcher^24^; see McGetrick and Range^7^ for a review).

Domestic dogs are a particularly interesting case in the study of inequity aversion. They exhibit a basic form of inequity aversion in that they refuse to continue participating in a task with an experimenter if unrewarded in the presence of a rewarded partner. Range et al.^25^ first demonstrated this using the so-called “paw task”. In this study, pairs of dogs were repeatedly asked for the paw, one after the other and received identical or different rewards to each other, each time they gave the paw, depending on the condition. Unlike in studies with capuchin monkeys^6^, chimpanzees^26^, and many other non-human animal species, there was no effect of unequal reward distributions in terms of reward quality; subjects did not refuse to participate in the experiment if the partner received a better quality reward for performing the same task. However, subjects discontinued significantly earlier when *unrewarded* in the presence of a rewarded partner compared with when both they and the partner received rewards.

Refusing to continue complying with the experimenter earlier in this “reward inequity” condition compared with the equity condition does not in itself indicate that dogs are inequity averse, however. The absence of reward could elicit non-compliance from subjects simply because they themselves are not being rewarded, rather than declining to participate based on social comparison of payoffs and the observed unequal reward distribution. To tease apart the effect of reward omission from the effect of the unequal reward distribution, Range et al.^25^ included an additional “no-reward” control condition in which the subject was unrewarded for providing its paw on each trial but no partner was present. Taking inspiration from critiques of primate inequity aversion studies^8,9^ relating to the presence and movement of food rewards, in this no-reward control condition Range et al.^25^ moved a piece of food towards the partner’s unoccupied position on each trial, as though feeding a dog, before moving it back to the bowl. The aim of this movement from an experimental perspective was to control for the movement of food that occurs when feeding a partner in the inequity condition. Subjects gave up significantly earlier in the inequity condition than in this no-reward control condition, suggesting that the negative responses were due to inequity. The finding of inequity aversion has been replicated in at least five studies, demonstrating its robustness^27–31^.

It has recently been highlighted, however, that the aimless movement of food in the no-reward control condition may have been problematic^7^. From the dog’s perspective, observing a food reward being moved in the experimenter’s hand, in the absence of a clear goal or recipient, could have resulted in the perception that food rewards were being offered or were attainable. This perception that rewards were on offer or attainable in the no-reward control condition could have motivated dogs to comply with the experimenter for longer than they otherwise might have. Interestingly, it has been shown that humans integrate information about both reward value and perceived attainability when making decisions in cognitive tasks^32^. It is conceivable that the significantly longer performance typically exhibited by dogs in the no-reward control compared with the inequity condition of these tasks may be due to differences in perceived reward attainability across conditions rather than differences based on social comparison of rewards.

Our aim in the current study was to test this perceived reward attainability hypothesis. We repeated the paw task of previous studies^25,27,29,31^, but we introduced two additional asocial conditions in which the partner dog’s position was occupied by a box containing an opening into which rewards could be placed on each trial. The first of these conditions was the equity—box condition in which the subject was rewarded on each trial for giving the paw and a reward was also inserted into the box on each trial. The second was the inequity—box condition in which the subject did not receive a reward each time it gave the paw, but a reward was inserted into the box on each trial. We expected to replicate findings from previous studies whereby subjects give up earlier in the inequity condition than both the equity condition and no-reward condition. We predicted that, if the hypothesis of perceived reward attainability is true, then no difference in subjects’ performance would emerge between the inequity condition with a dog as a partner and the inequity—box condition in which rewards are inserted into the box in a manner matching the feeding of a partner. Additionally, we predicted, subjects would give up earlier in the inequity—box condition than in the standard no-reward condition in which the partner’s position is unoccupied.

## Methods

### Ethical approval

All procedures were approved by the Ethics and Animal Welfare Committee of the University of Veterinary Medicine, Vienna (ethical protocol nos.: ETK-21/10/2017; ETK-01/11/2018) in accordance with the University’s guidelines for Good Scientific Practice. Additionally, dog owners were required to sign a consent form prior to participation in the study.

### Subjects

Twenty pet dogs were tested in this study (14 f; 6 m; mean age ± SD = 4.70 ± 2.49 years; see Table 1 for details of the sample) in a within-subjects design. These were largely recruited as pairs from the same household. None of these dogs had taken part in a paw task study before. Criteria for inclusion in the study comprised prior training in how to give the paw on command, how to sit on command, and being at least one year of age. Training to sit and give the paw had been carried out either by the owner or a trainer and not by the experimenter. The extent or nature of the participants’ training to sit and give the paw on command was not documented.

**Table 1:** Details of participants in the study, including dyad, sex, age, and breed.

Subject	Dyad	Sex	Age (years)	Breed
1	1	M	2.7	Landseer
2	2	F	7.0	Spitz
3	2	F	5.0	Spitz
4	3	F	8.0	Cocker Spaniel
5	4	F	3.1	Australian Shepherd
6	5	F	9.0	Border Collie
7	6	F	2.5	Boxer
8	7	F	2.6	Newfoundland Dog
9	7	M	4.1	Bernese Mountain Dog
10	6	M	5.3	Golden Retriever
11	4	F	4.1	Shetland Sheepdog
12	8	F	7.4	Parson Russell Terrier
13	9	F	4.8	Mixed breed
14	9	F	8.6*	Mixed breed
15	10	M	3.7	Mixed breed
16	10	M	7.8	Mixed breed
17	1	F	5.2	Australian Shepherd
18	3	F	1.0	Husky
19	8	F	7.7	Mixed breed
20	5	M	1.2	Border Collie

*Estimate; exact age not known.

In the experiment, for each dyad, the subject was the individual of interest; however, roles were reversed throughout the study such that the partner was also tested as a subject. Sixteen different subjects were tested beforehand in a pilot version of this study (see supplementary information for more details).

### Paw task

#### General setup and procedure

The general setup and procedure largely matched that of previous paw task studies^25,27^, applying a within-subjects design. All tests were carried out in a room (7 m × 6 m) at the “Clever Dog Lab” of the Messerli Research Institute, located at the University of Veterinary Medicine, Vienna, Austria. Four cameras, with one positioned close to each corner of the room, were used to record each experimental session. Three female experimenters in total carried out experimental sessions but each subject had all their sessions with one of these.

When both dogs in the dyad (i.e. the subject and the partner) were present, they were seated approximately 0.5–1 m apart. They were both leashed at a wall on leashes approximately 1.5 m in length. A wooden block (60 cm × 10 cm × 10 cm) separated the subjects. The owner stood in between the two dogs, against the wall, and remained passive unless interference was required (e.g. if aggressive interactions between the dogs occurred, or if the leash got wrapped around the dog). The experimenter knelt in front of the two dogs with a bowl (approximately 30 cm in diameter) containing small pieces of food. The subject and partner were asked for the paw alternately, by the experimenter. The partner always received a small food reward for giving the paw, but whether the subject was rewarded, depended on the experimental condition. In asocial conditions no live partner was present. The dogs could not reach the food in the bowl. The rewards used in this study were small pieces of sausage (approximately 1 cm × 1 cm × 1 cm), except for two dyads (i.e. four subjects) for which the owner provided their own dry-food treats. Each time the experimenter moved a food reward (e.g. to feed a dog; see also the test conditions below), she raised the reward above the bowl, roughly at, or above, the dogs’ eye level, so that both dogs (or only the subject if the subject was alone) could see the reward being distributed. The experimenter then moved the reward in the required direction or to the required destination. A second experimenter sat approximately two metres away, noting whether the subject gave the paw, and the number of paw and sit commands issued, on each trial.

Two experimental sessions were conducted per day, per subject, with a 10-min break in between. A session consisted of 30 trials for the subject if alone. If the subject and partner were both present, a session consisted of 30 trials for the subject and partner each, one after the other, alternating between the subject and partner after each trial, always beginning with the partner. Sessions were shorter if one of two possible termination criteria were reached on a particular trial with the subject: repeatedly refusing to give the paw or repeatedly refusing to sit (see below).

A trial consisted of a dog being asked to give the paw until it complied or until a termination criterion was reached. The request for the paw was performed with an outstretched palm combined with the German command for giving the paw, “Pfote!”. If a dog failed to give its paw on the first paw command (after approximately 2–3 s), the command was repeated, with the flat palm of the hand being presented each time. The command was repeated up to ten times, calling the dog’s name on approximately the fifth repetition. If the dog successfully gave its paw, the trial was considered complete and the experimenter moved onto the next trial. The subject received a food reward for completing a trial unless the experimental condition required that it not receive a reward. If the subject failed to give its paw after eleven sequential paw commands, one possible termination criterion was reached and the entire session was terminated. The count of paw commands was applied within each trial only; thus, if a subject successfully gave its paw before the maximum number of paw commands was reached on a particular trial, the criterion started afresh on the next trial. The partner dog always received a food reward for successfully completing a trial. The strict number of paw commands was not applied to the partner as we required the partner to always give the paw; otherwise, we could not continue the session with the subject. If the partner dog refused to give the paw, the experimenter was permitted to lure it into giving the paw using a food treat.

The second termination criterion pertained to sit commands. Before the dogs were asked for the paw, they were required to sit. If a dog was not already sitting, it was requested to sit with an outstretched finger and the German command “Sitz!”. If the dog did not sit in response to this command, the command was repeated a further nine times with the subject’s name being called after the fifth repetition. If the dog changed position majorly (e.g. from a lying to a standing position) in the middle of this sequence of commands, the sequence of ten sit commands began again. If the subject did not comply after ten sit commands in a row, while in the same position (e.g. standing or lying), the session was terminated. As with the paw commands above, the count of sit commands was applied within each trial only. If a partner dog refused to sit after ten commands, the owner was asked to interfere and request the dog to sit, as the session could not be continued, and therefore no clear count could be obtained for the subject. The sit criterion was typically not applied strictly on the first trial of a session; if the subject or partner did not sit in response to requests, the owner was asked to interfere so that the session could begin appropriately.

#### Conditions

One session was conducted per condition per subject (unless otherwise stated). Six conditions were performed in this study (see Table 2). Two social conditions were performed as in previous studies: the equity condition and the inequity condition, here referred to as the “equity—dog” and “inequity—dog” conditions, respectively. In the equity—dog condition, both the subject and partner received a single food reward each time they gave the paw. In the inequity—dog condition, the partner received a food reward each time it gave the paw, but the subject did not receive a reward for giving the paw.

**Table 2:** Conditions tested in the paw task.

Condition	Subject rewarded?	Partner type	Acronym in former studies
Equity—Dog	Yes	Dog	ET
Inequity—Dog	No	Dog	RI
Equity—Box	Yes	Box	–
Inequity—Box	No	Box	–
Equity—Empty Space	Yes	No partner	AC
Inequity—Empty Space	No	No partner	NR

Two novel asocial conditions were introduced in this study: the “equity—box” and “inequity—box” conditions. The partner dog was not present, but its position was occupied by a vertically standing cardboard box (60 cm × 26 cm × 26 cm; see supplementary information, Fig. S1). The box had a circular opening located centrally towards its upper end. A plastic bottle cap from which the top had been cut out, was inserted and glued in this opening. This allowed the top half of a commercial plastic bottle to be screwed into the opening. Food rewards could be inserted into the opening in the box and would collect within the bottle-half inside the box.

The equity—box condition was similar to the equity—dog condition. The subject received a reward each time it gave the paw and, on each trial, a piece of food was moved towards the box and inserted into the circular opening, thereby matching the feeding of the partner dog that occurs in social conditions. The inequity—box condition largely matched the inequity—dog condition, as the subject did not receive a reward for giving the paw. However, on each trial, as in the equity—box condition, a food reward was inserted into the opening of the box.

**Figure 1:**
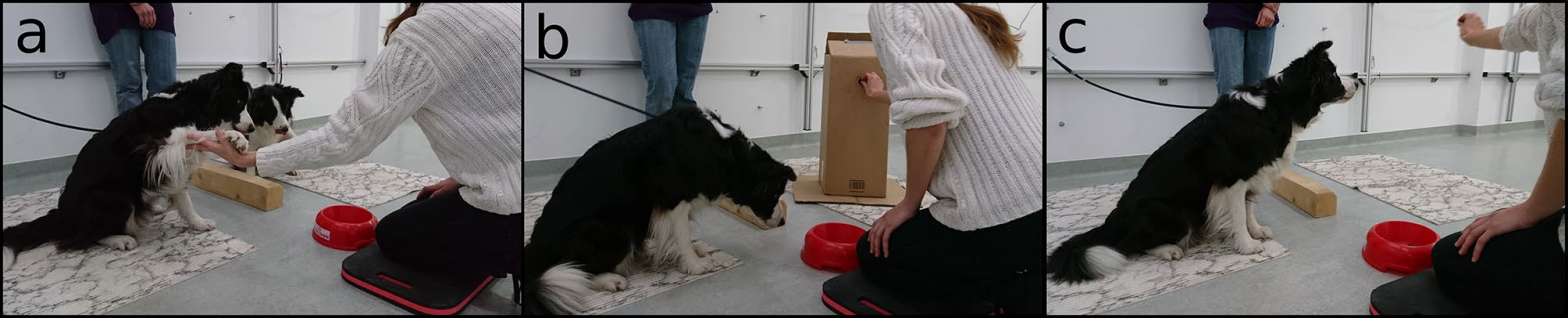
Paw task setup. Paw task setup with a conspecific (**a**) and the box (**b**) as partners, and with no partner (i.e. empty space) (**c**).

The final two conditions included in this study were also both asocial conditions. The partner dog was not present but its position remained unoccupied (see Fig. 1 for images of conditions with different partner types). Here, these are referred to as the “equity—empty space” condition and the “inequity—empty space” condition. In the equity—empty space condition, the subject received a reward each time it gave the paw, and a food reward was also lifted above the bowl, moved towards the partner’s unoccupied position (as though feeding a dog) and then moved back to the food bowl, on each trial. In the inequity—empty space condition, the subject did not receive a reward for giving the paw but, again, a food reward was moved towards the unoccupied partner’s position as though feeding a dog, and was then moved back to the food bowl.

Subjects were tested with the same partner type on a given day (i.e. dog, box, or no partner) with the equity condition always preceding the inequity condition. This particular order (i.e. equity followed by inequity) was chosen to match many of the previous inequity studies in which the *equity*—empty space condition always preceded the *inequity*—empty space condition^25,27,29,33^. Although this structure could have resulted in the subjects forming an expectation of reward in the first condition, which would later be violated, this would have been consistent across the unrewarded conditions, thereby allowing us to tease apart such reward schedule effects from the effect of partner type.

Both dogs in each dyad were tested as subjects. After one individual had completed two conditions as a subject on a particular test day, the other individual in the dyad was tested in two conditions as the subject. The first subject to be tested on a given day was tested in two asocial conditions (i.e. either the box or empty space conditions). Given that no partner was required in the asocial conditions, this meant that the subjects did not participate as a rewarded partner before being tested as a subject on the same day. The sequence in which each subject experienced the three possible test days (i.e. with the conspecific partner, with the box, and with no partner [empty space]) was, otherwise, random. Thus, the two dogs in a dyad could have been tested in the same asocial conditions as each other on the same test day or in different conditions.

For the social conditions, the experimenter began the session with the partner (i.e. asking the partner for the paw and feeding the partner); however, for the asocial conditions, the experimenter generally began each session with the subject (i.e. a reward was typically not deposited in the box before asking the subject for the paw).

### Behaviour coding

Behaviour coding was performed using Solomon coder (version beta 17.03.22^34^). We coded the number of trials on which the subject gave the paw. We also coded the number of paw and sit commands issued to the subject by the experimenter as the number of commands issued per trial has been used as an additional indicator of resistance to comply, with more commands being required in the inequity—dog condition than the inequity—empty space condition in previous studies^25,27^. The values obtained from this behaviour coding were used for the final analysis. For cases in which videos were unavailable due to technical malfunctions, results from a score sheet, manually prepared during the session by a second experimenter, were used. Interobserver reliability was assessed by comparing the counts obtained by video coding with the counts from the score sheets. This was performed for 20% of test sessions. Interobserver reliability was excellent (no. of times the paw was given: ICC = 1, n_observations_ = 24, *p* < 0.001; no. of paw and sit commands: ICC = 0.944, n_observations_ = 24, *p* < 0.001).

Seven sessions ended too early due to an incorrect count e.g. too few paw commands or too few sit commands (equity—dog × 1; inequity—dog × 2; inequity—box × 3; equity—empty space × 1). For these subjects, the stricter, incorrect count was applied to all conditions prior to analysis. This did not influence the results in any case.

Two observations were excluded from the analysis (inequity—dog × 1; inequity—box × 1) as the count was zero indicating the subject had not experienced the condition-specific reward distribution and, therefore, their response was unrelated to the experimental condition. A third observation was excluded as the session ended too early, at the request of the owner (inequity—empty space). A fourth session also ended early, at the request of the owner; this observation was not excluded from the analysis as it was an observation from an equity—dog condition and the count at the point at which the session ended was already considerably higher than in the inequity—dog condition, which is an important comparison.

### Statistical analysis

#### Number of times the subjects gave the paw (latency to give up)

To analyse the effect of condition on the number of times the subjects gave the paw (or the latency to give up), we fitted a Cox proportional hazards mixed effects regression model. The response variable included the number of trials completed by the subject and whether the event of “giving up” occurred (i.e. subjects who gave the paw on 30 trials did not give up but a count lower than 30 meant the subject gave up).

We included fixed effects of “rewarded” (i.e. whether the subject was rewarded or not), “partner” (i.e. the type of partner, which was either a dog, the box, or no partner [empty space]), and an interaction between these two factors, with the interaction being the main term of interest. To control for its potential effect, we included test day order (i.e. whether a particular condition occurred on the first, second, or third test day for each subject) as an additional fixed effect. We included random intercept effects of subject (i.e. the identity of the dog) and dyad (i.e. the identity of the subject-partner pairing). Random slopes of “partner”, “rewarded”, and test day order were included within both random effects with no correlations between random slopes and random intercepts. As an overall test of the effect of the interaction between the factors “rewarded” and “partner” we conducted a full-null model comparison, based on a likelihood ratio test. The null model lacked the interaction between “rewarded” and “partner” but was otherwise identical to the full model. A more detailed description of the analysis can be found in the supplementary information.

#### Number of paw and sit commands issued per trial

To analyse the effect of condition on the sum of the number of paw and sit commands issued per trial, we fitted a Generalized Linear Mixed Effects Model (GLMM) with a negative binomial distribution. The response variable comprised the total number of paw commands issued plus the total number of sit commands issued. We included the same fixed and random effects as above but retained correlations among the random slopes and random intercepts. To account for the differing number of trials completed across subjects, as an offset term we included the log of the number of trials on which the subject gave the paw. As above, we conducted a full-null model comparison to test the overall effect of the interaction between “rewarded” and “partner”. A more detailed description of the analysis can be found in the supplementary information.

## Results

### Number of times the subjects gave the paw (latency to give up)

**Figure 2:**
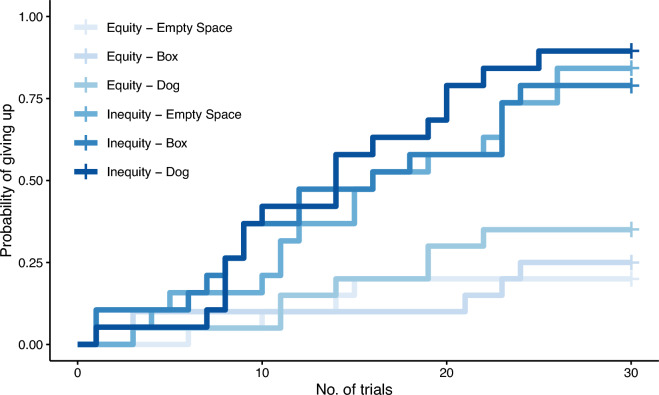
Probability of giving up across trials in each condition of the paw task. Equity—Empty Space, N = 20; Equity—Box, N = 20; Equity—Dog, N = 20; Inequity—Empty Space, N = 19; Inequity—Box, N = 19; Inequity—Dog, N = 19.

Overall, no significant interaction between the factors “rewarded” and “partner” was detected in the model assessing subjects’ latency to give up (i.e. the number of times they gave the paw; full-null model comparison: χ^2^ = 0.8292, *df* = 2, *p* = 0.661; see Fig. 2).

### Number of times the subjects gave the paw (inequity averse subjects)

The lack of a significant interaction between the fixed effects “rewarded” and “partner” means that there were no condition-specific differences that were independent of a “reward” effect (i.e. whether the partner was rewarded or not) or a “partner” effect (i.e. the type of partner present). In order to replicate the effect of inequity aversion observed in previous studies, we required a significant interaction between “rewarded” and “partner”, whereby subjects give up significantly earlier in the inequity—dog condition than both the equity—dog and inequity—empty space conditions. These criteria were not met; therefore, we failed to replicate the finding of inequity aversion observed in previous studies with dogs. The ability to test our hypothesis was dependent on having subjects that display inequity aversion as in previous studies. Therefore, we selected the subjects (N = 10) for which the response in the inequity—dog condition was lower than that in the inequity—empty space condition and thus showed aversion to inequity (based on the comparison used in previous studies). This was 50% of the original sample of dogs. We then compared the response in the inequity—dog condition with that in the inequity—box condition for these subjects. We also compared the response in the inequity—empty space condition with that in the inequity—box condition. Both comparisons were conducted using a two-sided Wilcoxon signed-ranks test.

**Figure 3:**
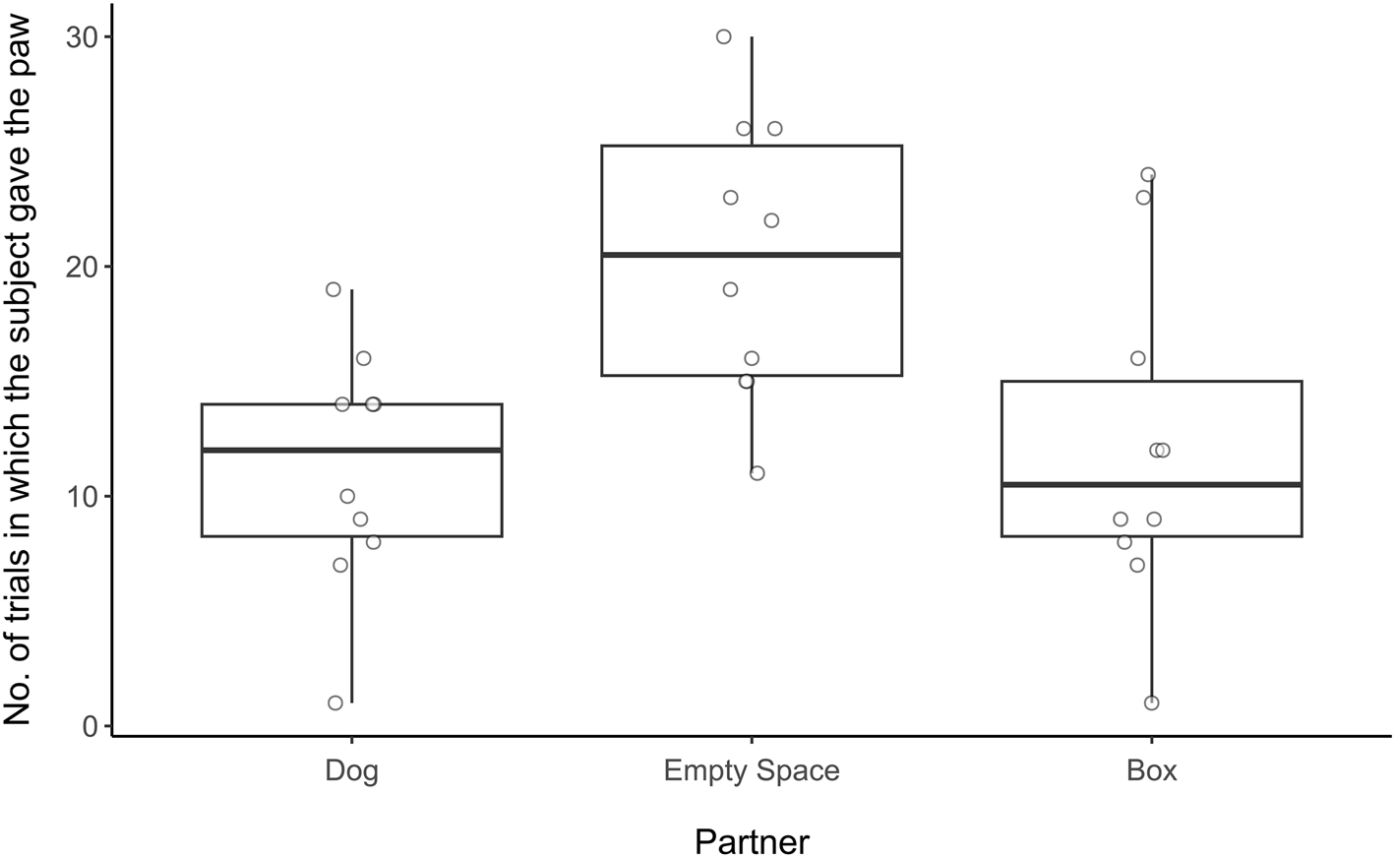
Number of trials in which the paw was given in each unrewarded condition. Boxes display the interquartile range, black horizontal bars represent the median, whiskers represent the range of data points within 1.5 times the interquartile range from the upper and lower hinge, and circles represent individual data points.

There was no significant difference in response between the inequity—dog condition and the inequity—box condition (Wilcoxon signed-ranks test: z = − 0.7158, *p* = 0.508). However, the subjects gave the paw significantly fewer times in the inequity—box condition compared with the inequity—empty space condition (Wilcoxon signed-ranks test: z = 2.5532, *p* = 0.0078; see Fig. 3). The order in which conditions were experienced across these ten inequity averse subjects appears to be random (see Table 3).

**Table 3:** Details of the 10 inequity averse subjects.

Subject	Dyad	Experimenter	Day 1	Day 2	Day 3	Sex	Age (years)	Breed
4	3	C	Box	Dog	Empty Space	F	8.0	Cocker Spaniel
6	5	B	Empty Space	Box	Dog	F	9.0	Border Collie
8	7	A	Empty Space	Dog	Box	F	2.6	Newfoundland Dog
10	6	A	Empty Space	Dog	Box	M	5.3	Golden Retriever
12	8	C	Box	Empty Space	Dog	F	7.4	Parson Russell Terrier
13	9	C	Box	Empty Space	Dog	F	4.8	Mixed breed
15	10	A	Empty Space	Box	Dog	M	3.7	Mixed breed
17	1	A	Dog	Box	Empty Space	F	5.2	Australian Shepherd
18	3	C	Dog	Box	Empty Space	F	1.0	Husky
19	8	C	Dog	Empty Space	Box	F	7.7	Mixed breed

Dyad identity, experimenter identity, the order in which the subjects experienced the conditions, as well as the sex, age, and breed of these subjects are included.

### Effect of age and sex on the probability of being inequity averse

To identify the factors that may have distinguished the inequity averse individuals from others in the overall sample, we analysed the effect of age and sex on the probability of being inequity averse. Neither sex nor age had a significant effect on the probability of being inequity averse (full-null model comparison: χ^2^ = 1.3075, *df* = 2, *p* = 0.520). The effect of breed was not analysed as this was almost equivalent to subject identity. A more detailed description of the analysis can be found in the supplementary information.

### Number of paw and sit commands issued per trial

Overall, no significant interaction between the factors “rewarded” and “partner” was detected in the model analysing the sum of the number of paw and sit commands issued per trial for the full sample of dogs (full-null model comparison: χ^2^ = 0.4255, *df* = 2, *p* = 0.808; see Fig. S5). For the ten inequity averse subjects, pairwise comparisons of the number of commands issued per trial indicate no significant difference between any of the unrewarded conditions (Wilcoxon signed-ranks test: inequity—dog vs inequity—empty space: z = 1.376, p = 0.193; inequity—dog versus inequity—box: z = − 0.2548, p = 0.846; inequity—empty space versus inequity—box: -1.58, *p* = 0.131; see Fig. S6).

## Discussion

Overall, no evidence for inequity aversion was obtained with the complete sample of dogs tested in this study. However, for the subset of subjects that displayed the standard response to inequity, performance was significantly lower in the inequity—box than inequity—empty space condition, but no difference was observed between the inequity—dog condition and the inequity—box condition. These results match our predictions and, therefore, support the hypothesis that dogs’ response in the inequity—empty space (or no-reward control) condition of such studies is inflated by perceptions of reward attainability.

The results of the current study do not indicate whether or not dogs are actually inequity averse, but rather demonstrate that giving up in inequity tasks does not necessarily reflect inequity aversion. Three lines of evidence from previous studies support the notion that dogs are inequity averse. First, in a food sharing test conducted immediately after each condition of the paw task, compared with the equity condition a shorter duration of co-feeding was observed after the inequity condition and a “quality inequity” condition in which the subject and partner received rewards of different quality^27^. Second, subjects spent significantly less time in proximity to their conspecific partner and took significantly longer to approach the experimenter after the inequity condition than after the equity condition^27^ (see also Essler et al.^28^). Third, in a “social control” condition in which both the subject and the partner were present and were required to perform the necessary task without reward, with a sample of 14 subjects Range et al.^25^ observed a trend whereby subjects gave up earlier (Wilcoxon signed-ranks test: n = 10 [4 ties], T +  = 10.0, *p* = 0.08) and required significantly more commands in the inequity condition than in this social control condition. Similarly, with a sample of 14 subjects, Romero et al.^30^ observed a significantly lower performance in the reward inequity condition than the social control condition (GLMM: z = − 2.765, *p* = 0.015). Thus, although we have presented support for perceived attainability influencing dogs’ performance in the inequity—empty space control, there is still evidence that dogs are inequity averse but that the empty space control over-exaggerates this effect and is, therefore, inappropriate for comparison with the inequity condition.

Although it seems unlikely, it is worth questioning whether the subjects in our study perceived the box as though it were a social agent. If subjects did perceive the box as a social agent, this would mean their negative response to being unrewarded in the presence of the rewarded box may constitute genuine inequity aversion. Humans are known to attribute animacy and intentionality to non-living geometric shapes based on movement patterns (a phenomenon referred to as perceptual animacy^35,36^). Recent studies suggest that dogs may similarly ascribe some form of animacy to moving shapes or objects^37,38^. The box in our study remained motionless for the most part during the experiment; therefore, it is implausible that subjects perceived the box as being animate based on movement cues. However, factors other than movement such as eyes^39,40^ or a face^41,42^ can also influence humans’ perception of animacy. It is tempting to speculate that observing an inanimate partner being fed could also stimulate perceptions of that inanimate partner’s animacy. Cook et al.^43^ recently observed different levels of amygdala activity in aggressive dogs when observing their owner feeding a fake dog compared with when their owner placed food in a bucket. This result provides some evidence that placing food in an inanimate object is insufficient to elicit perceptions of animacy, whereas inserting food into a fake dog is sufficient. It is, however, unclear whether the physical form and facial features of the fake dog, as opposed to the observation of the feeding of a life-like model, explain the subjects’ response. In our study, further to any possible perception of animacy, it is possible that each individual subject’s previous experience of retrieving food from inanimate objects such as boxes could have influenced their perception of the attainability of rewards. Although we did not document such previous experience in the main sample used in this study, for the sample in the pilot study (see supplementary information) experience of hunting or nosework did not influence the number of times subjects gave the paw in the inequity—box condition.

One unexpected finding of our study, which requires explanation, is the absence of inequity aversion in the complete sample of dogs tested. It is unclear what factors might have influenced the expression of inequity aversion. Our results indicate that age and sex did not influence the probability of being grouped in the inequity averse subsample. Breed is also unlikely to have had an influence, as a previous study demonstrated that both cooperative worker and independent worker breeds display the standard response to inequity^31^, and our sample also included a variety of dog breeds. To the best of our knowledge, this is the first paw task study that has not observed a negative response to inequity with the complete sample of dogs. It is worth noting, however, that two versions of a paradigmatically similar “buzzer task” study with dogs failed to observe inequity aversion^33^. Furthermore, Horowitz^44^ and McAuliffe^45^, each with a unique task, found no evidence for inequity aversion in dogs. Thus, the finding of inequity aversion may not be as robust as would seem when focusing on the paw task alone. Further studies on the processes and factors influencing dogs’ responses in such tasks will undoubtedly help to explain these divergent findings.

The lack of replication of previous findings with our sample may call into question the generalizability of our results with the “inequity averse” subsample. However, it is important also to acknowledge that the results of our pilot study support those of the main study in that no difference was observed between the inequity—dog condition and the inequity—box condition (though empty space conditions were not included; see supplementary information for details). This pilot study was conducted by one male experimenter who did not conduct any of the sessions included in the main study and involved 16 subjects that were also not part of the main study. These results are, therefore, independent from the main study. Regardless of the generalizability of our findings, the perceived reward attainability hypothesis remains a valid hypothesis to explain previous findings of inequity aversion in the paw task, and additional controls such as the box conditions included here are required in such studies to rule this out.

Despite demonstrating that the no-reward control (inequity—empty space) condition of previous studies could have generated inflated counts, it remains unclear exactly what mechanism governs responses in the inequity—dog and inequity—box conditions. It seems plausible that the low paw counts are simply responses to the lack of reinforcement across trials. However, rather than simply omitting reinforcement in these conditions, the perceived reward attainability for the dogs may have been lowered relative to the inequity—empty space condition and such low perceived attainability may influence their decision. Therefore, it is possible that the low counts in the inequity condition of inequity aversion studies could be driven by the perceived reward attainability being relatively low, rather than reflecting a basic response to not receiving a reward. Additional investigations could also determine whether the disappearance of the food as opposed to the goal-directed movement of the food by the experimenter influences the dogs’ behaviour in this experimental context.

Although we propose perceived reward attainability as an alternative to inequity aversion as an explanation for results in previous studies with dogs, it is important to acknowledge that it is not always clear what constitutes inequity aversion. Based on Fehr and Schmidt’s^3^ initial conception and definition of inequity aversion as the resistance to inequitable outcomes, different characterizations of inequity aversion are possible. The first characterization is as a behavioural outcome^4^: individuals achieve an effective negative response to, or avoidance of, inequity, regardless of the underlying proximate mechanism (hereafter, “functional” inequity aversion). Numerous authors, notwithstanding^1,3,4,9,10,15^, appear to imply or assume specific cognitive and emotional properties associated with inequity aversion, leading to a proximate level characterization of inequity aversion^46^: individuals recognize differences in quality or quantity between their own payoff and that of a social partner and this underlies their response (hereafter, “true” inequity aversion). Inequity aversion tasks with non-human animals, including this study, typically test implicitly for *true* inequity aversion. However, as also alluded to by Brosnan and Bshary^4^ *functional* inequity aversion could conceivably come about independently of *true* inequity aversion. Thus, although the results of our study suggest that dogs’ responses in these inequity tasks do not reflect *true* inequity aversion, it is still plausible that dogs could achieve a similar functional outcome in social situations.

## Conclusion

In conclusion, our results suggest that the longer duration of paw-giving in the no-reward control condition in inequity studies with dogs is driven by perceptions of reward attainability. More specifically, movement of food in the absence of a partner in the no-reward control condition increases perceptions of reward attainability for subjects, leading them to work for significantly longer than they do in the inequity condition. Thus, the comparison between the no-reward condition and the inequity condition may create the illusion that dogs are giving up in the inequity condition due to true inequity aversion.

## Supplementary Information

Supplementary Information 1.

Supplementary Information 2.

## Data Availability

The data analysed in this study are included in the supplementary materials.
