# Supplementary material for: Perceived reward attainability may underlie dogs’ responses in inequity paradigms
Source: Sci Rep. 2023 Jul 26;13:12066. doi: 10.1038/s41598-023-38836-w (PMC10372141; doi:10.1038/s41598-023-38836-w)
Supplement: Supplementary file 2 — Supplementary Information 2. [file 41598_2023_38836_MOESM2_ESM.pdf]

Supplementary Information:

Perceived reward attainability may underlie dogs' responses in inequity paradigms

Jim McGetrick[1,2]*, Hugo Peters[1], Anna D. J. Korath[1], Romana Feitsch[1], Susanne Siegmann[1], Friederike Range[1]

[1]Domestication Lab, Konrad Lorenz Institute of Ethology, Department of Interdisciplinary Life Sciences, University of Veterinary Medicine, Vienna, Dörfles 48, A-2115 Ernstbrunn, Austria

[2]Institute of Animal Welfare Science, University of Veterinary Medicine, Veterinärplatz 1, 1210 Vienna, Austria

*Corresponding author: Jim.McGetrick@vetmeduni.ac.at

ORCID:	Jim McGetrick:	0000-0001-5468-934X
	Anna D.J. Korath:	0000-0002-6408-4157
	Susanne Siegmann:	0000-0003-2671-5066
	Friederike Range:	0000-0003-3127-5536

Table of Contents

Main Study ... 3
Methods ... 3
Statistical Analysis ... 4
Results ... 11
Pilot study ... 13
Methods ... 13
Subjects ... 13
Procedure and conditions ... 13
Statistical Analysis ... 13
Results ... 17
Number of times the subjects gave the paw (latency to give up) ... 17
Effect of beginning the study as a subject or a partner ... 18
Effect of experience of hunting or nosework ... 18
Number of paw and sit commands issued per trial ... 19
References ... 20

Main Study

Methods

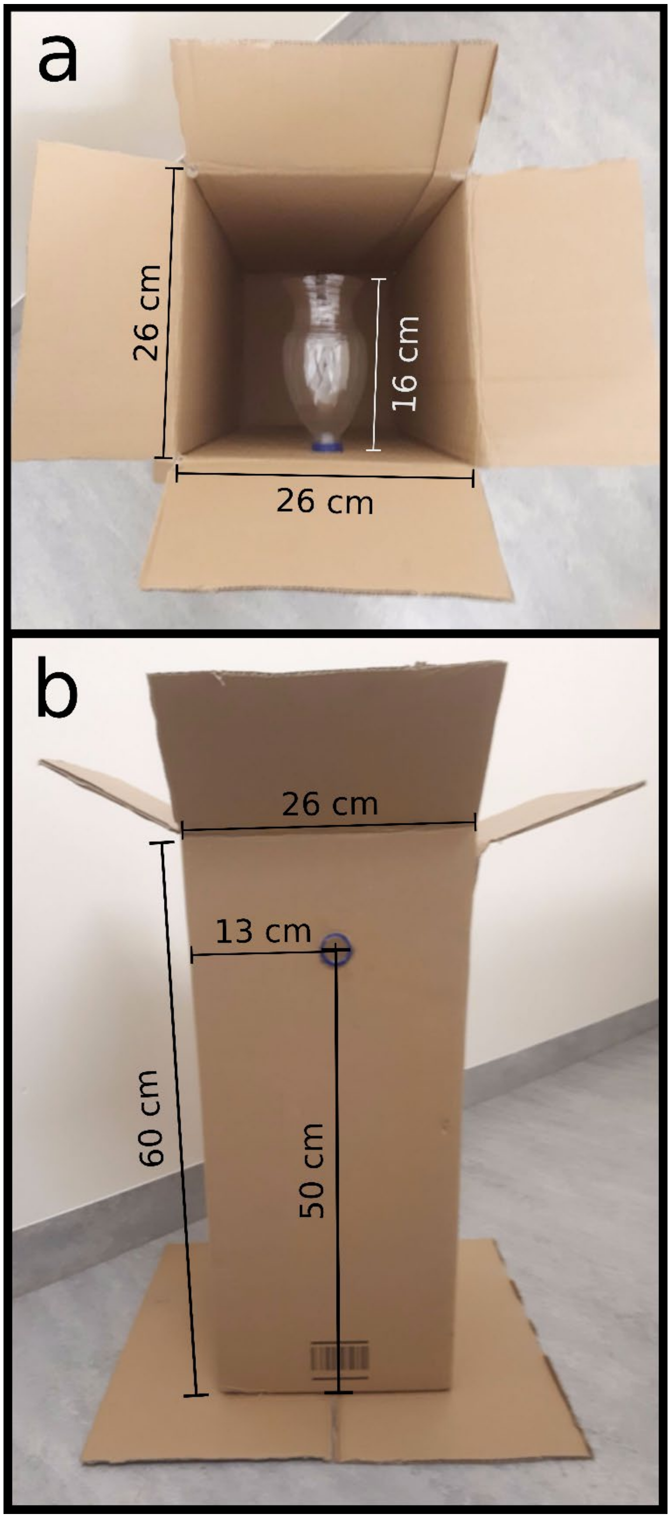

Fig. S1 Appearance and dimensions of the box used. a, top view; b, front view.

Statistical Analysis

All models were fitted in R (versions 3.6.2 – 4.3.0[1]). The packages and functions used in each case are given below. Random slopes were identified, and overdispersion and model stability were assessed, where necessary, using functions kindly provided by Roger Mundry. Boxplots were created using the R package ggplot2 (versions 3.3.2 – 3.4.2[2]).

No. of times the subjects gave the paw (latency to give up)

To analyse the effect of condition on the number of times the subjects gave the paw, we fitted a Cox proportional hazards mixed effects regression model. The response variable included the number of trials completed by the subject and whether the event of "giving up" occurred (i.e. subjects who gave the paw on 30 trials did not give up but a count lower than 30 meant the subject gave up).

We included fixed effects of "rewarded" (i.e. whether the subject was rewarded or not), "partner" (i.e. the type of partner, which was either a dog, the box, or no partner [empty space]), and an interaction between these two factors, with the interaction being the main term of interest. To control for its potential effect, we included test day order (i.e. whether a particular condition occurred on the first, second, or third test day for each subject) as an additional fixed effect. We included random intercept effects of subject (i.e. the identity of the dog) and dyad (i.e. the identity of the subject-partner pairing).

In order to avoid overconfidence with regards to the precision of the estimates for fixed effects, and to keep type *I* error rate at the nominal level of 5%, we included almost all identifiable random slopes[3,4] which included the random slopes of "rewarded", "partner", and test day order within the random effects of subject and dyad.

The model could only be fitted by excluding the correlations between the random slopes and random intercept; therefore, we excluded these correlations. We fitted the model using the function "coxme" in the package "coxme" (version 2.2-16[5]). Prior to fitting the model, we z-transformed test day order to a mean of zero and a standard deviation of one to allow for an easier interpretation of results. The factors "rewarded" and "partner" were dummy coded and centred for inclusion as random slopes.

We assessed model stability at the level of the estimated coefficients and standard deviations by excluding the levels of the random effects one at a time[6]. This revealed the model generally to be of acceptable stability (see **Fig. S2**).

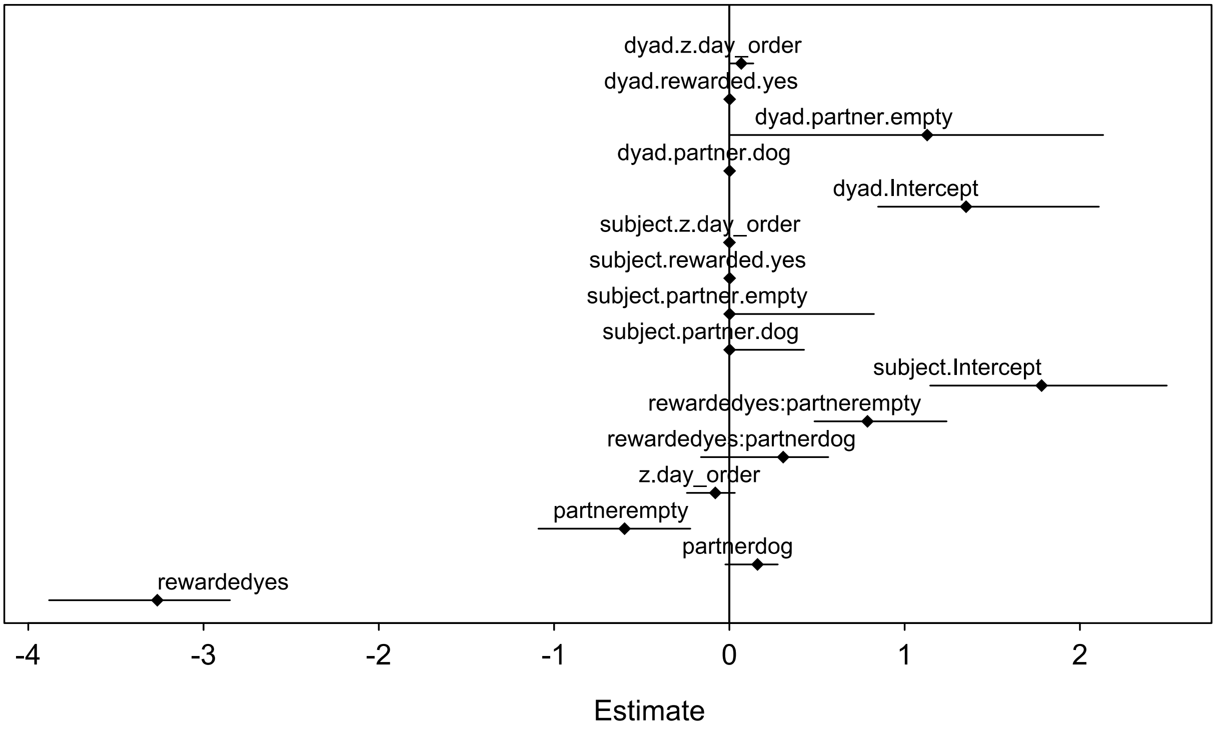

Fig. S2 Model stability plot. Model stability plot for Cox regression model analysing subjects' latency to discontinue with the task. This plot represents the range of model estimates for each term in the model when the levels of the random effects were excluded one at a time. Names beginning with "dyad" and "subject" are the random effects: the term after the first full stop is either the random intercept or a random slope.

As an overall test of the effect of the interaction between the factors "rewarded" and "partner" we conducted a full-null model comparison[7], aiming at avoiding cryptic multiple testing, whereby the null model lacked the interaction between "rewarded" and "partner" but was otherwise identical to the full model. This comparison was based on a likelihood ratio test[8] using the R function "anova" and setting the "test" argument to "Chisq". The sample for this model included a total of 117 observations across 20 subjects and 10 dyads.

A cumulative incidence plot representing the probability of discontinuing with the task across conditions and trials was created using the package "survminer" (version 0.4.9[9]).

To assess interobserver reliability, we calculated the intraclass correlation coefficient using the function “icc” in the package “irr” (version 0.84.1[10]), setting the “model” argument to “twoway” and the “type” argument to “consistency”.

Inequity averse individuals only

Pairwise comparisons were conducted using a Wilcoxon signed-ranks test, specifically using the “wilcoxsign_test” function in the package “coin” (version 1.3-1[11,12]) and by setting the “distribution” argument to “exact” and the “alternative” argument to “two.sided”.

Effect of age and sex on the probability of being inequity averse

To analyse the effect of age and sex on the probability of being inequity averse, we fitted a Generalized Linear Mixed Model (GLMM) with a binomial error distribution and a logit link function[13,14] (0, not inequity averse; 1, inequity averse).

We included condition, age and sex as fixed effects and dyad as a random intercept effect. No random slopes were included. Age was z-transformed to a mean of zero and a standard deviation of one prior to fitting the model. We fitted the model using the function “glmer” from the package “lme4” (version 1.1-33[15]). Assessment of model stability, carried out as above, produced extreme estimates (see **Fig. S3**). This appeared to be due to one specific dyad (dyad 6). To test for the effects of age and sex we conducted a full-null model comparison as above. The null model lacked the fixed effects of age and sex. The sample for this model included a total of 20 observations across 10 subjects.

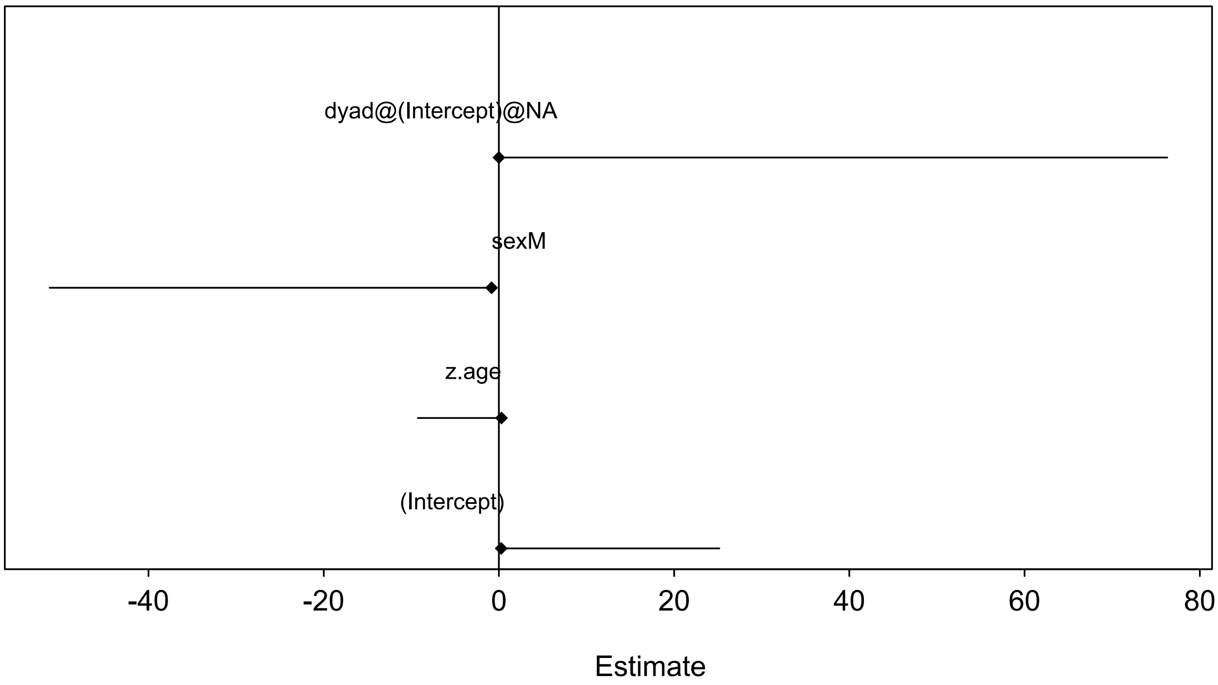

Fig. S3 Model stability plot. Model stability plot for the probability of being inequity averse, representing the range of model estimates for each term in the model when the levels of dyad were excluded one at a time.

Number of paw and sit commands issued per trial

To analyse the effect of condition on the number of commands issued per trial, we initially fitted a GLMM with a Poisson error distribution[13,14]. The response variable comprised the total number of paw commands issued plus the total number of sit commands issued.

We included fixed effects of "rewarded", "partner", and an interaction between these two factors, with the interaction being the main term of interest. To control for its potential effect, we included test day order as an additional fixed effect. To account for the differing number of trials completed across subjects, as an offset term[14] we included the log of the number of trials on which the subject gave the paw.

We included random intercept effects of subject and dyad. All theoretically identifiable random slopes were included[3,4]; these were the random slopes of "rewarded", "partner", and test day order within the random effects of both subject and dyad. "Rewarded" and "partner" were manually dummy coded and centred for inclusion as random slopes.

We fitted the model using the function "glmer" from the package "lme4" (version 1.1-23[15]). Prior to fitting the model, we z-transformed test day order to a mean of zero and a standard deviation of one. This model was overdispersed (dispersion parameter: 1.825). To deal with overdispersion we included an observation level random effect. However, this resulted in an underdispersed model (dispersion parameter: 0.45).

Given these issues with overdispersion and underdispersion, we decided to fit a GLMM with a negative binomial distribution, dropping the observation level random effect. We fitted this model using the function "glmer.nb" from the "lme4" package (version 1.1-23[15]).

Assessment of model stability was carried out as above. The model was of good stability for the fixed effects, random intercepts, and random slopes; model stability for correlations among random slopes and random intercepts was acceptable (see **Fig. S4**; for four models out of 30 in the model stability analysis, the iteration limit was reached).

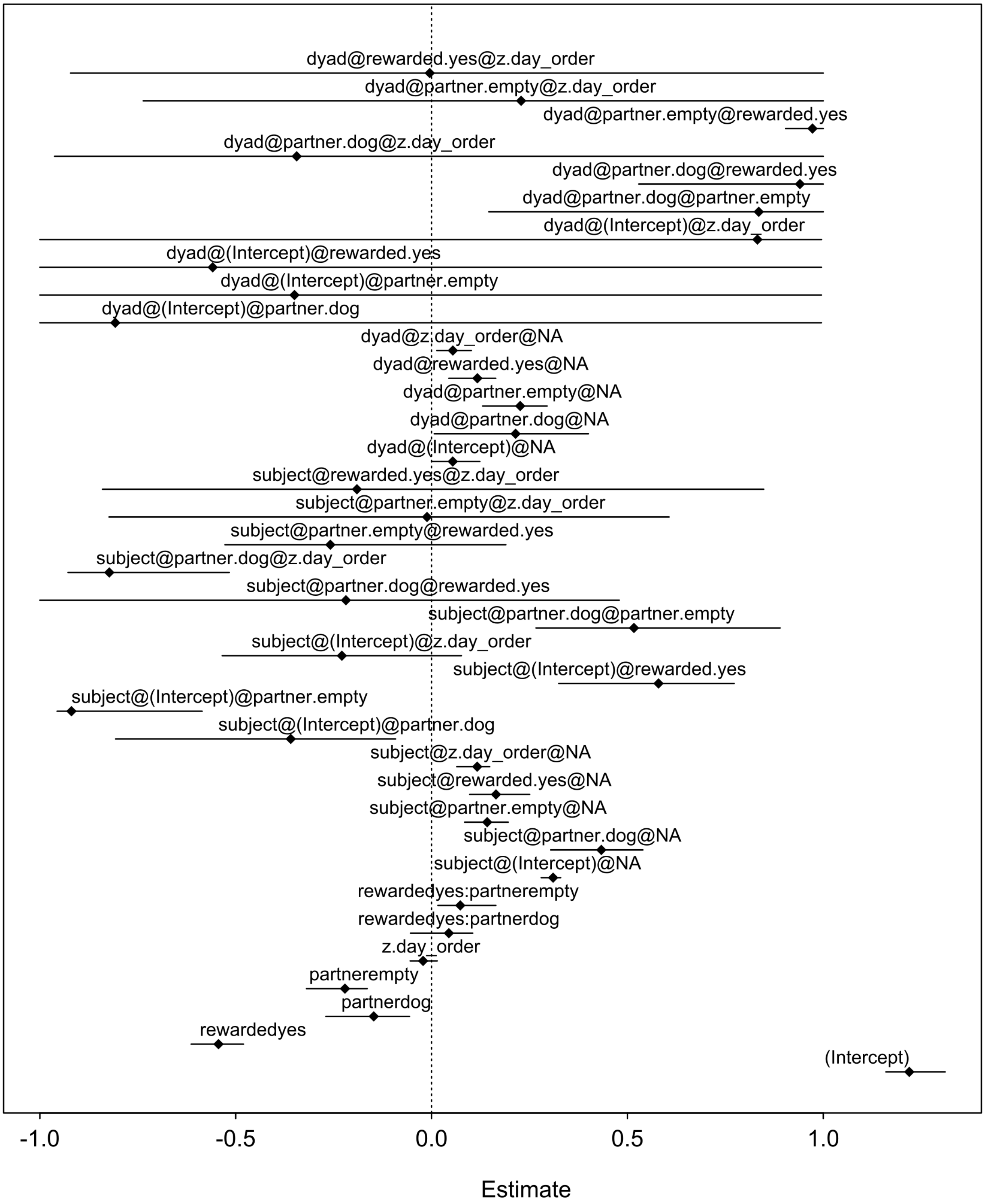

Fig. S4 Model stability plot. Model stability plot for the number of commands issued to subjects per trial, representing the range of model estimates for each term in the model when the levels of the random effects were excluded one at a time. Names including an ampersat symbol (@) refer to random effects: the first term in the name is the grouping variable, the term after the first ampersat is either a random intercept (indicated by "(Intercept)") or a random slope; if there is also a name after the second ampersat, this effect represents a correlation within the random effect.

As an overall test of the effect of condition, we conducted a full-null model comparison, as above[7]. The null model lacked the interaction between “rewarded” and “partner”. The sample for this model included a total of 117 observations across 20 subjects and 10 dyads. Interobserver reliability for the total number of commands issued was excellent (ICC = 0.944, $n_{observations} = 24$, $p < 0.001$).

Inequity averse individuals only

Pairwise comparisons were conducted using a Wilcoxon signed-ranks test, specifically using the “wilcoxsign_test” function in the package “coin” (version 1.3-1[11,12]) and by setting the “distribution” argument to “exact” and the “alternative” argument to “two.sided”.

Results

Results for the number of times the subject gave the paw (latency to give up), the effect of age and sex on the probability of being inequity averse, and the number of paw and sit commands issued per trial, are presented in the main article.

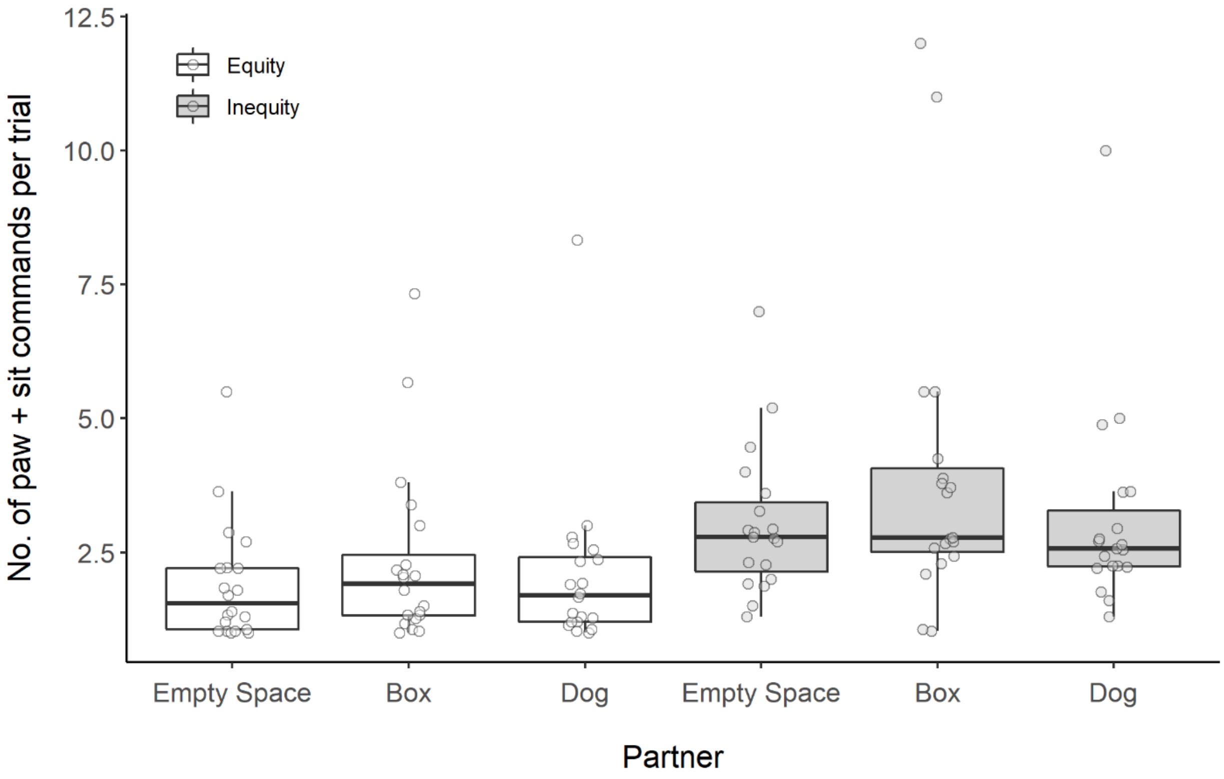

Fig. S5 Number of paw and sit commands issued per trial in each condition of the paw task. Boxes display the interquartile range, black horizontal bars represent the median, whiskers represent the range of data points within 1.5 times the interquartile range from the upper and lower hinge, and circles represent individual data points. Equity – Empty Space, N = 20; Equity – Box, N = 20; Equity – Dog, N = 20; Inequity – Empty Space, N = 19; Inequity – Box, N = 19; Inequity – Dog, N = 19.

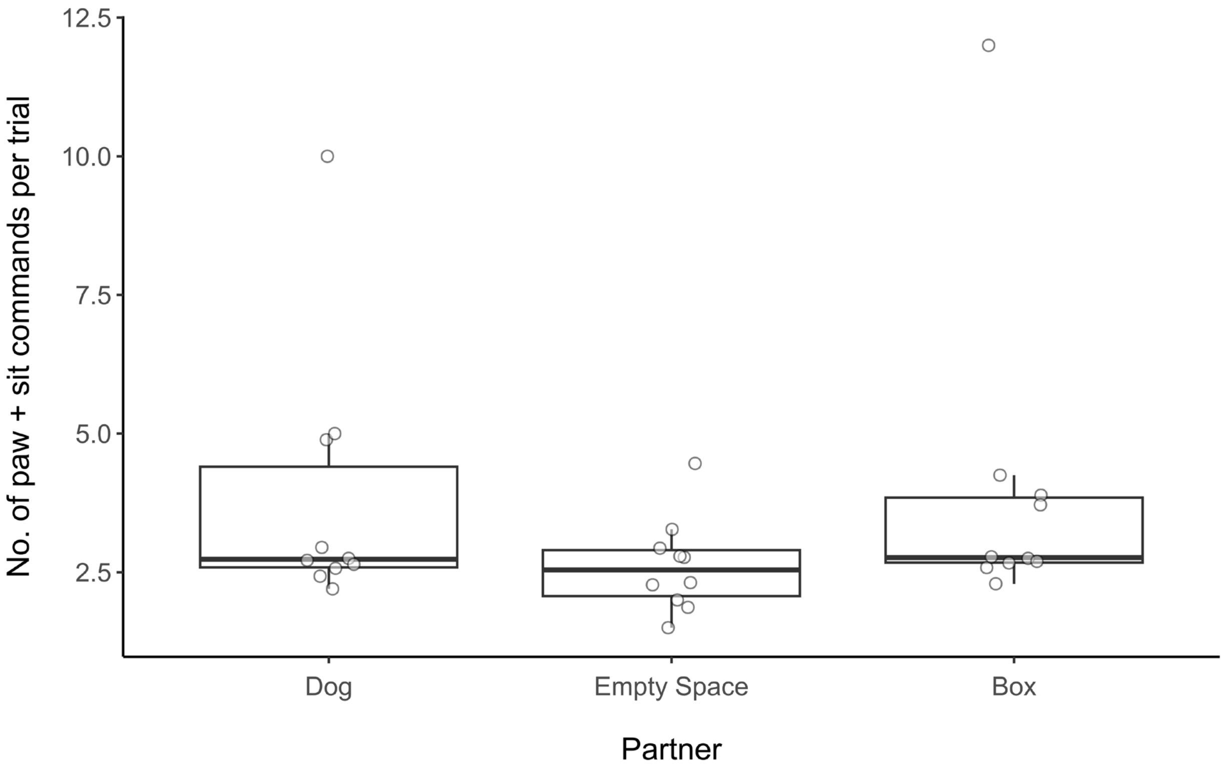

Fig. S6 Number of paw and sit commands issued per trial in unrewarded conditions of the paw task. Only the 10 inequity averse subjects are included. Boxes display the interquartile range, black horizontal bars represent the median, whiskers represent the range of data points within 1.5 times the interquartile range from the upper and lower hinge, and circles represent individual data points.

Pilot study

An initial pilot study was carried out prior to the study presented in the main article. The methods resembled those for the main study, except for differences identified below.

Methods

Subjects

Sixteen pet dogs were tested in this study (6 f; 10 m; mean age ± SD = 5.69 ± 3.42 years). These were sixteen different dogs to those tested in the main study.

Procedure and conditions

These dogs were tested by one male experimenter and were only tested in the two social conditions with the dog as a partner (equity – dog and inequity – dog) and the two asocial conditions with the box as a partner (equity – box and inequity – box; see **Table 2** in the main manuscript). In most cases, both dogs in the dyad were included in the study as subjects. After the first subject in a dyad had completed all four conditions, the roles were reversed. The order in which the social and asocial conditions were experienced was counterbalanced across all subjects. An error in performance of the procedure occurred in a total of four sessions; thus, these sessions were repeated on a third test day. Three of the subjects were recruited from the same household; therefore, one dog played the role of the partner twice.

Videos were coded by one experimenter. Interobserver reliability was assessed by comparing counts obtained by video coding with the counts from score sheets which were manually scored during the test sessions. This was performed for 20% of test sessions. Interobserver reliability was excellent (no. of times the paw was given: ICC = 0.999, $n_{observations} = 13$, $p < 0.001$; no. of paw and sit commands: ICC = 0.996, $n_{observations} = 13$, $p < 0.001$).

Statistical Analysis

No. of times the subjects gave the paw (latency to give up)

The number of times the subjects gave the paw was analysed as above. The sample for this model included a total of 68 observations across 16 subjects and 8 dyads.

The model was of acceptable stability generally with the exception of the interaction between "rewarded" and "partner" which produced an extreme range and the random slope of "rewarded" within the random effect of "subject" (see **Fig. S7**).

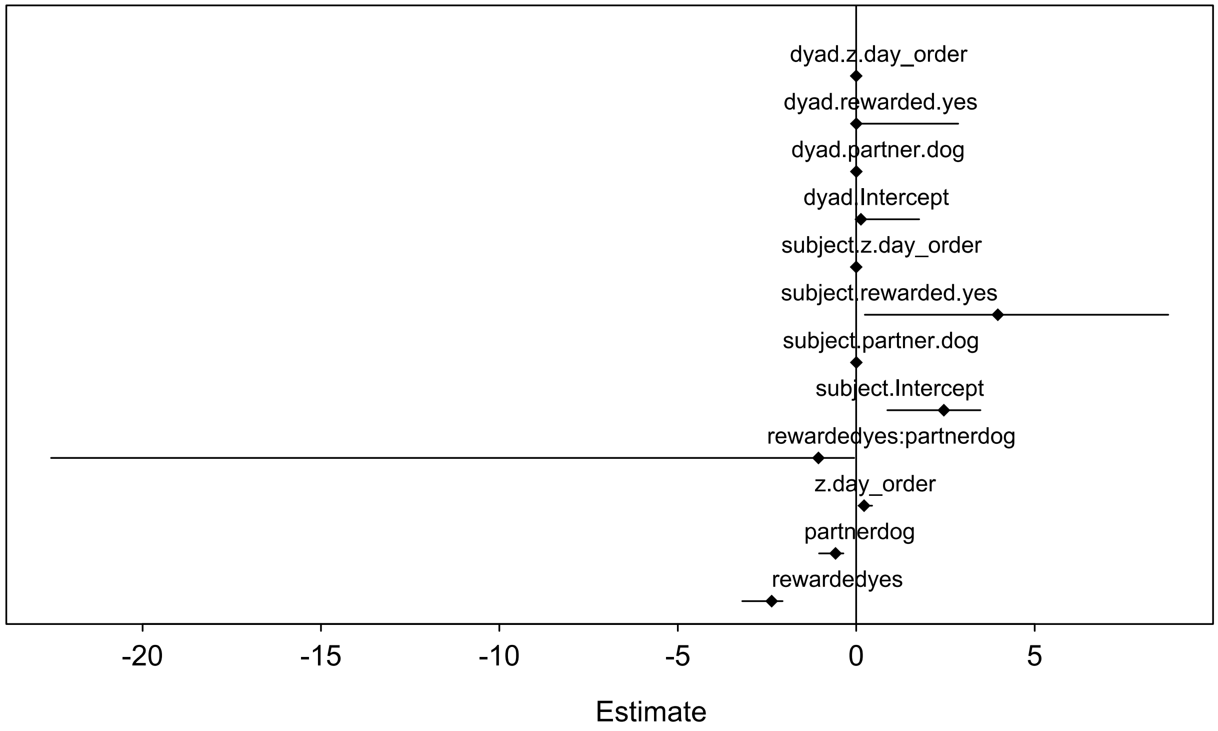

Fig. S7 Model stability plot. Model stability plot for Cox regression model analysing subjects' latency to discontinue with the task. This plot represents the range of model estimates for each term in the model when the levels of the random effects were excluded one at a time. Names beginning with "dyad" and "subject" are the random effects: the term after the first full stop is either the random intercept or a random slope.

Effect of beginning the study as a subject or a partner

We analysed the effect of whether the subjects carried out all of their sessions as the subject first or began the study playing the role of the partner, using a Wilcoxon rank sum test using the "wilcox_test" function in the package "coin" (version 1.4-2[11,12]), setting the "distribution" argument to "exact" and the "alternative" argument to "two.sided".

Effect of experience of hunting or nosework

We also analysed the effect of previous experience with hunting or nosework on the number of times they gave the paw in the inequity – box condition. Subjects were divided into two categories: no experience of hunting or nosework and some experience of hunting or

nosework. This information was based on a questionnaire typically filled out by participants of studies at the Clever Dog Lab. We carried out a Wilcoxon rank sum test using the "wilcox_test" function in the package "coin" (version 1.4-2[11,12]), setting the "distribution" argument to "exact" and the "alternative" argument to "two.sided".

Number of paw and sit commands issued per trial

To analyse the effect of condition on the number of commands issued per trial, we fitted a GLMM[13,14] with a Poisson error distribution. The model had the same structure with regards to the response variable, fixed effects, random effects, and offset term as the model for the number of paw and sit commands in the main study. We fitted the model using the function "glmer" from the package "lme4" (version 1.1-23[15]). This model was overdispersed (dispersion parameter: 1.168) and one correlation between a random slope and random intercept was close to minus one indicating that it was unidentifiable. We removed the correlations between the random slopes and random intercepts. Overdispersion was no longer an issue (dispersion parameter = 0.870). The model was of acceptable stability, generally (see **Fig. S8**). The sample for this model included a total of 64 observations across 16 subjects and 8 dyads. Interobserver reliability for the total number of commands issued was excellent (ICC = 0.996, $n_{observations} = 13$, $p < 0.001$).

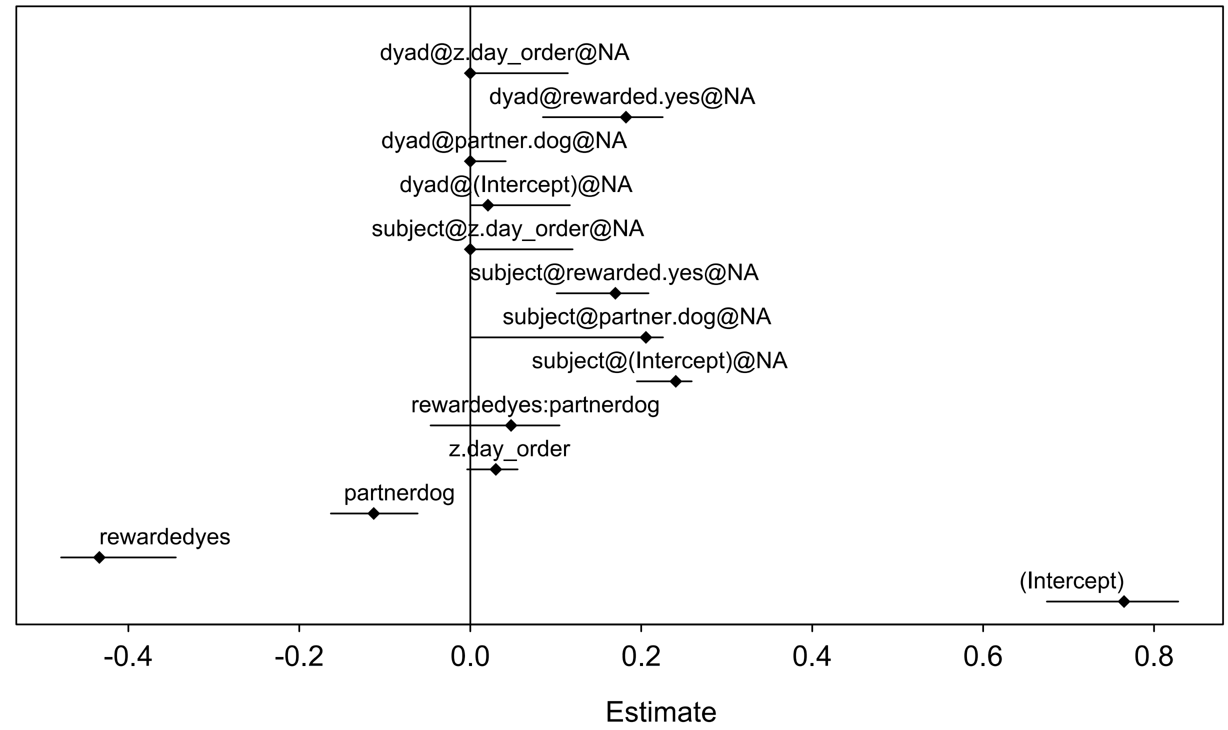

Fig. S8 Model stability plot. Model stability plot for the number of commands issued to subjects per trial, representing the range of model estimates for each term in the model when the the levels of the random effects were excluded one at a time. Names including an ampersat symbol (@) refer to random effects: the first term in the name is the grouping variable, the term after the first ampersat is either a random intercept (indicated by “(Intercept)”) or a random slope.

Results

Number of times the subjects gave the paw (latency to give up)

Overall, no significant interaction between the factors “rewarded” and “partner” was detected in the model assessing subjects’ latency to give up (i.e. the number of times they gave the paw; full-null model comparison: $\chi^2 = 0.6261$, $df = 1$, $P = 0.429$; see **Fig. S9**).

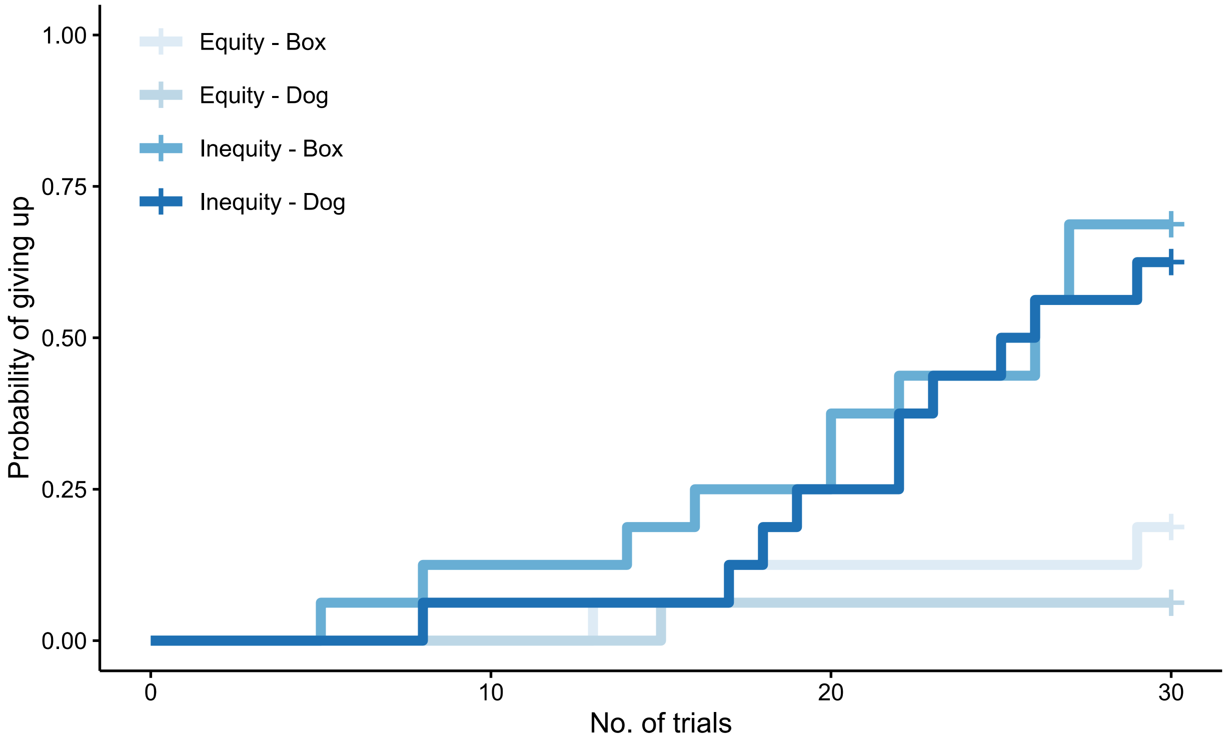

Fig. S9 Probability of giving up across trials in each condition of the paw task. All conditions, N = 16.

Effect of beginning the study as a subject or a partner

There was an effect of the subject's first role on the number of times they gave the paw (Wilcoxon rank sum test: $z = 2.4697$, $p = 0.013$). Individuals that began the study playing the role of the subject gave the paw fewer times than those that began by playing the role of partner and later became subjects (see **Fig. S10**).

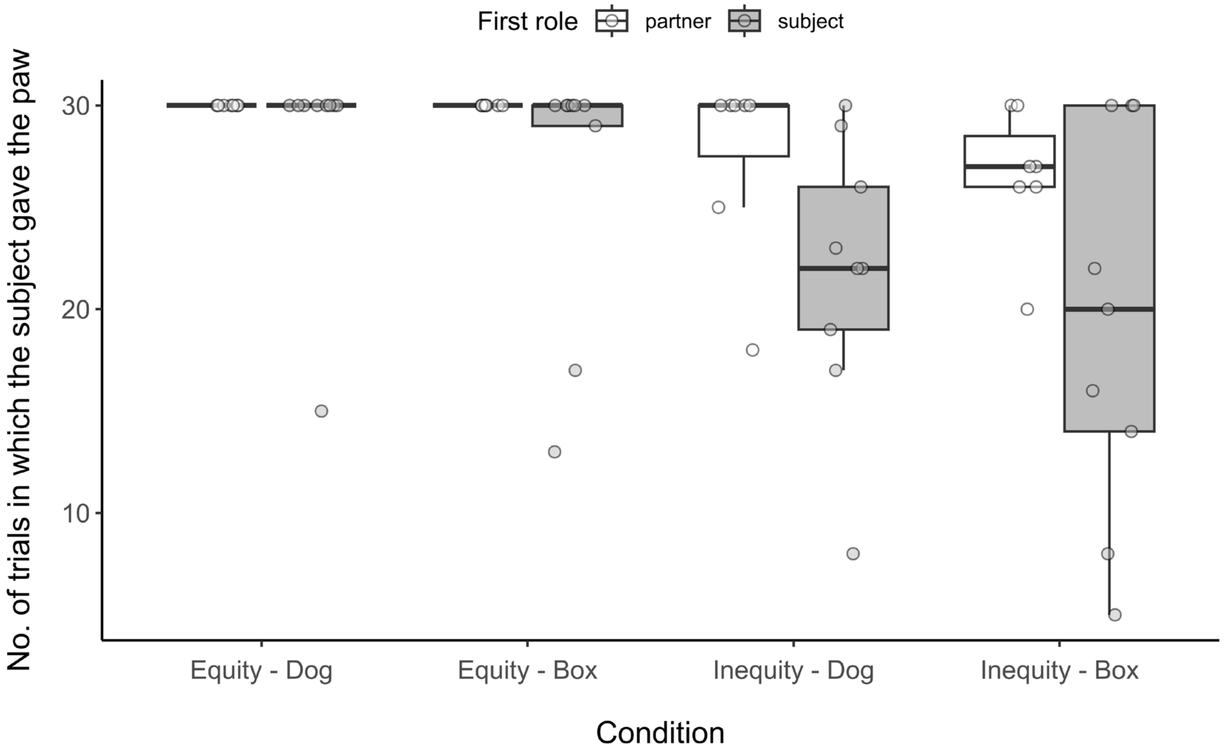

Fig. S10 Number of trials in which the paw was given in each condition. Data is separated based on whether the subjects began the study playing the role of the partner (white) or the subject (grey). Boxes display the interquartile range, black horizontal bars represent the median, whiskers represent the range of data points within 1.5 times the interquartile range from the upper and lower hinge, and circles represent individual data points.

Effect of experience of hunting or nosework

There was no effect of experience of hunting or nosework on the number of trials in which the subjects gave the paw in the inequity – box condition (Wilcoxon rank sum test: $z = -0.7000$, $p = 0.514$).

Number of paw and sit commands issued per trial

Overall, no significant interaction between the factors "rewarded" and "partner" was detected in the model analysing the number of commands issued per trial (full-null model comparison: $\chi^2 = 0.3729$, $df = 1$, $P = 0.541$; see **Fig. S11**).

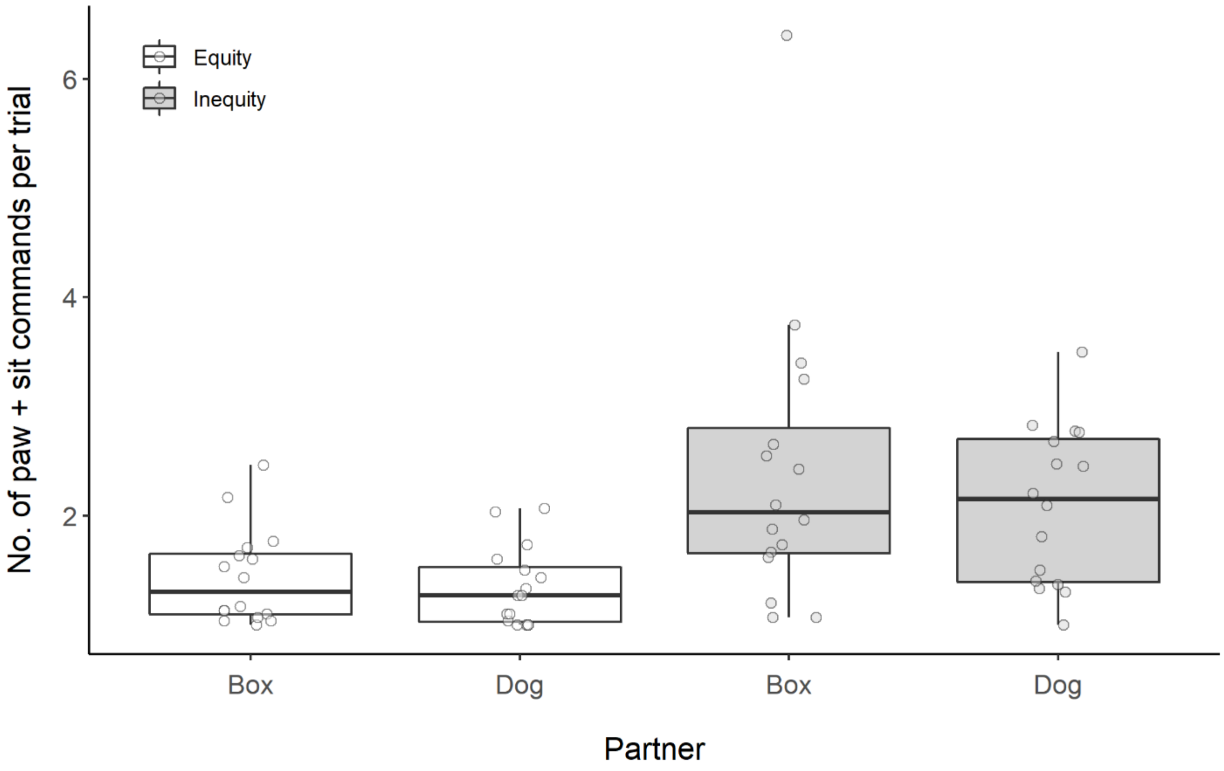

Fig. S11 Number of paw and sit commands issued per trial in each condition of the paw task. Boxes display the interquartile range, black horizontal bars represent the median, whiskers represent the range of data points within 1.5 times the interquartile range from the upper and lower hinge, and circles represent individual data points. All conditions, N = 16.

References

1. R Core Team. R: A language and environment for statistical computing. R Foundation for Statistical Computing, Vienna, Austria. https://www.r-project.org/ (2023).
2. Wickham, H. *ggplot2: Elegant Graphics for Data Analysis*. (Springer-Verlag New York, 2016).
3. Schielzeth, H. & Forstmeier, W. Conclusions beyond support: overconfident estimates in mixed models. *Behav. Ecol.* **20**, 416–420 (2009).
4. Barr, D. J., Levy, R., Scheepers, C. & Tily, H. J. Random effects structure for confirmatory hypothesis testing: Keep it maximal. *J. Mem. Lang.* **68**, 255–278 (2013).
5. Therneau, T. M. coxme: Mixed Effects Cox Models. R package version 2.2-16, https://cran.r-project.org/package=coxme (2020).
6. Nieuwenhuis, R., te Grotenhuis, M. & Pelzer, B. influence.ME: Tools for Detecting Influential Data in Mixed Effects Models. *R J.* **4**, 38–47 (2012).
7. Forstmeier, W. & Schielzeth, H. Cryptic multiple hypotheses testing in linear models: Overestimated effect sizes and the winner's curse. *Behav. Ecol. Sociobiol.* **65**, 47–55 (2011).
8. Dobson, A. J. *An Introduction to Generalized Linear Models*. (Chapman & Hall/CRC, 2002).
9. Kassambara, A., Kosinski, M. & Biecek, P. survminer: Drawing Survival Curves using 'ggplot2'. R package version 0.4.9, https://cran.r-project.org/package=survminer (2021).
10. Gamer, M., Lemon, J., Fellows, I. & Singh, P. irr: Various Coefficients of Interrater Reliability and Agreement. R package version 0.84.1, https://CRAN.R-project.org/package=irr (2019).
11. Hothorn, T., Hornik, K., Van De Wiel, M. A. & Zeileis, A. A lego system for conditional inference. *Am. Stat.* **60**, 257–263 (2006).
12. Hothorn, T., Hornik, K., Wiel, M. A. van de & Zeileis, A. Implementing a Class of Permutation Tests: The **coin** Package. *J. Stat. Softw.* **28**, 1–23 (2008).
13. Baayen, R. H. *Analyzing linguistic data: A practical introduction to statistics using R*. (Cambridge University Press, 2008).
14. McCullagh, P. & Nelder, J. A. *Generalized Linear Models*. (Chapman & Hall/CRC, 1989).
15. Bates, D., Mächler, M., Bolker, B. & Walker, S. Fitting Linear Mixed-Effects Models Using **lme4**. *J. Stat. Softw.* **67**, 1–48 (2015).
